# AVATAR Therapy for Distressing Voices: A Comprehensive Account of Therapeutic Targets

**DOI:** 10.1093/schbul/sbaa061

**Published:** 2020-05-06

**Authors:** Thomas Ward, Mar Rus-Calafell, Zeyana Ramadhan, Olga Soumelidou, Miriam Fornells-Ambrojo, Philippa Garety, Tom K J Craig

**Affiliations:** 1 Department of Psychology, Institute of Psychiatry, Psychology and Neuroscience, King’s College London, London, UK; 2 South London and Maudsley NHS Foundation Trust, London, UK; 3 Mental Health Research and Treatment Center, Faculty of Psychology, Ruhr-Universität Bochum, Bochum, Germany; 4 Department of Health Service and Population Research, Institute of Psychiatry, Psychology and Neuroscience, King’s College London, London, UK; 5 Research Department of Clinical, Educational and Health Psychology, University College London, London, UK

**Keywords:** auditory hallucinations, voices, relational therapy, digital health, cognitive models

## Abstract

AVATAR therapy represents an effective new way of working with distressing voices based on face-to-face dialogue between the person and a digital representation (avatar) of their persecutory voice. To date, there has been no complete account of AVATAR therapy delivery. This article presents, for the first time, the full range of therapeutic targets along with information on acceptability and potential side effects. Interest in the approach is growing rapidly and this report acts as a necessary touchstone for future development.

## Introduction

A new wave of relational approaches has emerged in the context of psychosis, focusing on the interpersonal relationship between the voice-hearer and the voice.^1–3^ In AVATAR therapy,^1,4^ a novel therapeutic context allows “face-to-face” dialogue between the person and a digital representation matching the auditory characteristics and associated imagery of their main persecutory voice. The aim is for the voice-hearer to develop increased power and control, consistent with broader cognitive approaches.^5^ Building on pilot work,^1^ the recent first fully-powered trial^4^ of AVATAR therapy compared with an active control, found rapid and substantial reductions in voice frequency and distress (and associated omnipotence), with a post-therapy effect size of 0.8 suggesting that it is a more effective therapy for voices than current alternatives.

To date, AVATAR therapy has been described only in terms of broad treatment goals, specifically with respect to increased voice-hearer power, control, and self-esteem. A complete account of AVATAR therapy delivery is now crucial in developing our understanding of change in this unique therapeutic context.^6^

### Aim

This study aims to present, for the first time, a comprehensive account of AVATAR therapy including the full range of therapeutic targets, along with information on therapy acceptability and potential side effects.

### Method

TW (Therapy Lead) and TC (Principal Investigator) conducted a systematic case review of therapy completers (*n* = 53), drawing on detailed notes in standardized therapy booklets which recorded session-by-session therapy. Ten a priori therapeutic targets were identified from the AVATAR trial^4^ clinical manual. Each case was rated for “Full,” “Partial,” or “No” evidence of addressing one or more of these targets. Rare instances where ratings remained unclear were resolved through additional review of therapy letters and listening to audio-recordings of sessions. Data on acceptability are presented with regards to withdrawal.

### Results

Therapists were clinicians with prior experience of working within a cognitive- behavioral therapy approach for psychosis; training included 1–2 closely supervised pilot cases. Demographics and clinical descriptives are presented in Table 1.

**Table 1.** Demographics and Clinical Descriptives (*N* = 53 Therapy Completers)

Demographics and Clinical Descriptives	Percentage	Mean (SD)
Age		43.2 (10.3)
Length of illness (years)		21.3 (6.7)
Gender		
Male	77	
Female	23	
Ethnicity		
White British	32	
Black British/Caribbean	26	
Other	42	
Diagnosis		
Paranoid schizophrenia	79	
Schizoaffective disorder	11	
Other	10	
Number of sessions		
7 sessions	77	
8–10 sessions	21	
<7 sessions^a^	2	
Number of voices		
Single voice	23	
2–5 voices	56	
Unsure/many	21	
Voice mirrors past relationship?		
Clear evidence	66	
Possible	15	
No evidence	19	

^a^One person completed at session 3 reporting complete cessation of voices.

## AVATAR Therapy: Description and Therapeutic Targets

### Therapy Structure

AVATAR therapy has, to date, been delivered with people with a diagnosis of schizophrenia and related psychoses and “adequate” pharmacological treatment.^1,4^ Therapy on the recent trial^4^ comprised an initial session (assessment and avatar creation) followed by, typically, six weekly sessions (involving avatar dialogue). Fifty-three people completed therapy, more than 75% of whom attended the standard 7 sessions (Table 1). Therapy was most straightforward with a clear dominant voice (66%). While we did not identify any absolute contraindications, the therapy is potentially less indicated for individuals reporting diffuse nonpersonified voice phenomenology where these experiences cannot be represented clearly by a single avatar. For multiple voices, the focus was on the most distressing voice.

**Fig. 1.**
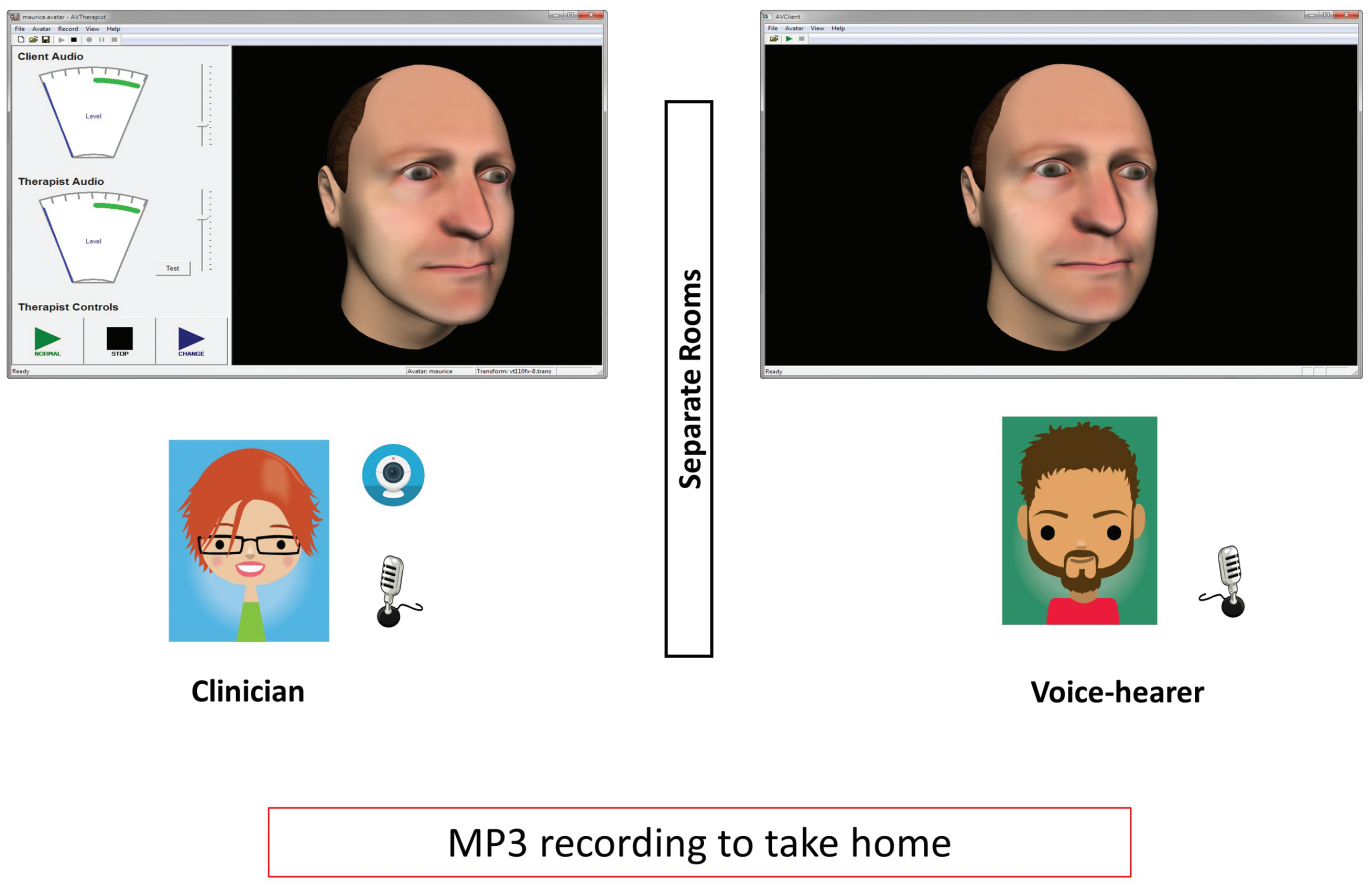
Set up for AVATAR therapy.

Each session had three parts, lasting approximately 60 min:

- Predialogue: review and agree the focus of the dialogue
- Active dialogue: (approximately) 5 min in early sessions, increasing to 10–15 min. Participant and therapist sat in separate rooms but in direct communication (Figure 1).
- Postdialogue: reflection on dialogue; a recording was provided.

### Therapy Phases

Active dialogue involved two phases:

#### Phase 1: Exposure and Assertiveness

Phase 1 (typically sessions 1 and 2) involved exposure to the avatar voicing verbatim content while the person was supported to respond assertively. This allowed processes of desensitization and involved expectancy violation and dropping safety behaviors; crucial for attenuating the conditioned fear response.^7^ Typical content (delivered through the voice-transformed avatar) included critical, abusive, and hostile comments. With the exception of commands to harm self/others, verbatim was used as reported. It was striking how voice-hearers related naturally to the avatar and maintained a clear separation between the therapist and avatar. Each dialogue ended on a “win” (eg, voice-hearer being supported to make a self-affirming statement), and exposure was typically associated with decreased anxiety over time. Toward the end of phase 1, the avatar adopted a conciliatory position; transition was carefully calibrated to mirror the voice-hearer’s increasing sense of power.^8^

#### Phase 2: Relational, Developmental and Emotional Processes

In addition to the continued focus on power, control, and autonomy, phase 2 followed a formulation incorporating autobiographical context, meaning-making, and experiences of trauma and powerlessness.^3,9^

#### Phase 2: Therapeutic Targets.

The 10 therapeutic targets (Table 2) are described below with illustrative examples (using anonymized first names):

**Table 2.** Ten Therapeutic Targets With Frequency of Use and Typical Examples

	Example			Use	
Theme	Context	Avatar	Person	Clear	Partial
Power and control	Voice-hearer delivers assertive statements (re)claiming power and control. Avatar concedes power.	“You are stronger than I thought”	“I’m not a victim anymore, I decide what I do now”; “You’ve been saying these things for years … I don’t believe you”	53 (100%)	—
Self-esteem/self-concept	Voice-hearer draws on a list (from a friend/relative) of personal qualities in dialogue with avatar.	“I say what you believe deep down … you are worthless, If this changes I’ll have nothing to say”	“I never believed someone could see me positively”; “I”m a good person and I don’t deserve punishment” [see Claire; text]	51 (96%)	1 (2%)
Maintenance processes	Identifying drivers of distress— inactivity, avoidance, tackling isolation/shyness, work on worry.	“It’s easy to control you when lock yourself away from others”	“I am going to be aware but not *over aware*.”; “I’ve started to go out more and meet people”	27 (51%)	4 (8%)
Working toward internal attribution	Voice related to “self”/ internal processes (eg, self- critical thoughts).	“I’m part of you. I’m here to keep you in line”	“So, if I was more confident you would have nothing to say”	14 (26%)	10 (19%)
Identity/social inclusion	Autobiographical disconnection from others. Rescripting experiences of racism/ discrimination in which voice-hearer has felt voiceless.	“I never considered the damage my words could cause”; “I’m listening now, what was it like for you?” [opening space]	“You need to educate yourself mate, get some knowledge”; “I’ve always been a fish out of water.. too white for school and too black when we moved [reflecting on dual heritage]”	27 (51%)	-
Compassion to voice	Shared experiences, understanding and forgiveness; positive function within the voice-hearer relationship	“I treat you this way to keep you on guard, you know the danger out there”	“We are just two black men in a hostile white world”; “I needed you in the past but I’m ready to take over now”	10 (19%)	3 (6%)
Experiential disengagement	Disengaging from avatar taunts or ego-dystonic content; practiced with avatar then used in daily life.	“I hate that man” “Look at that fat bitch/ mong” [provocative content”]	Focus on remaining cool and calm. Using neutral verbal response (“nothing to do with me”) or mindful silence.	19 (36%)	4 (8%)
Working with grief	Unresolved grief; resolution/ rescripting of conflicts; expression of love, understanding and forgiveness	“I couldn’t find the words when I was alive- but you’re a good man, I’m proud of you”	“You never understood that being kind was not my weakness, it was a strength” “[See Sid; text]	6 (11%)	5 (9%)
Working with trauma	Work on shame and self-blame. “Then-Now” discrimination.	“I never thought you would dare to stand up to me”	“I’m a grown woman…you can’t harm me anymore”; “It was not my fault”	19 (36%)	20 (38%)
Future focus	Moving forward (work, college, and relationships)	“You don’t need me running your life anymore…. What does the future hold for you?”	“It’s time for me to start living my life. Doing what I want…I’m in control”	45 (85%)	1 (2%)

### Power and Control

Enactment of the avatar allowed voice-power to be undermined “from within,” targeting beliefs about omnipotence, malevolence, and identity.^10^ The avatar was voiced as an entity that exaggerated its power, while the therapist encouraged the person to call the voice’s bluff (“you’ve been making threats for years … I don’t believe you”) thereby reclaiming power within the relationship.

### Self-esteem/Self-concept

Work on self-esteem was used explicitly for all but one person. The avatar often stated “I am only saying the negative things you believe about yourself…” foregrounding the role of self-concept. For some, increasing self-acceptance appeared to change their attribution of the voice (see below). Many brought a list of personal qualities from a “significant other” into the dialogue. The person thereby viewed themselves through the eyes of an “other,” a process of mentalization^11^ mirrored by the avatar’s changing view. For example, Claire presented with an entrenched sense of herself as evil and deserving punishment from her “demonic” voice. She experienced a “black line aura” surrounding her body; a signifier of intrinsic badness and toxicity. Claire felt shame regarding an intrusive thought during her mother’s final days (taken as evidence of her evil) and the fact that she missed the death (her voice emerged in this context). Claire used dialogue to voice the truth (“I am a good person, a loving mother and daughter”), facilitating shifts in emotional meaning. Over time, she reported increased power and control over the voice and the disappearance of the “black line.”

### Maintenance

In over half of cases, avatars named processes that maintained voice-distress,^12^ eg, social isolation and avoidance, inactivity, self-criticism, and hypervigilance, cueing discussion around making real-world change (“I can’t keep locking myself away and living in fear”).

### Working Toward Internal Attribution

An understanding that the voice may reflect a part of the self was a clear focus in 14 people (26%) (“I voice your deepest fears”) with more tentative work in a further 10 people (19%). This typically followed self-esteem work, requiring sensitivity to avoid invalidation.

### Identity/Social Inclusion

Voices often communicated the unacceptability and “otherness” of the hearer, delivering chronic shame.^13^ Chris experienced highly characterized “High-court Judges,” denouncing him for intrusive sexual thoughts (“You’re guilty, you know what you’ve done”). Through dialogue, Chris came to accept himself as a human being with sexual urges, the Head Judge acknowledging, ultimately, “there is no case to answer.” By the end of therapy, Chris had started dating for the first time in years.

### Compassion Toward the Voice

Compassion toward the voice was observed in around a quarter of people, with dialogs identifying positive voice functions, eg, “protection from a hostile world.” The avatar could also compassionately mirror experiences, facilitating empathic responses and problem-solving discussions about building new relationships. As with work on internal attribution, sensitivity to the potential for invalidation was crucial (see “Discussion” section).

### Experiential Disengagement

In just over one third of cases, the voice-hearer practiced disengagement from avatar taunts. This occurred where the voice-hearer was becoming drawn into repetitive arguments (“clinging” relationships^14^). The voice-hearer learned, experientially, how the avatar/voice provoked a response and practiced disengagement from the “tug-of-war.” AVATAR therapy shared common ground with mindfulness- and acceptance-based approaches in these exchanges.^15,16^

### Working With Grief

One person, “Sid,” heard the voice of his deceased father, a figure of terror and intimidation, in whose eyes he had felt “weak and small.” The avatar/ father had his eyes opened to what Sid was truly like as a man; “Being kind doesn’t make me weak” emerged as a key theme as the avatar transitioned to a father who “did not know how to express love.” Sid visited his father’s grave for the first time in many years to say “goodbye.” At the final session, and follow-up, Sid reported no longer hearing the voice.

### Working With Trauma

Some voice-content reflected phrases directly spoken by past abusers. For others, the link was thematic,^17^ eg, the phrase “I am going to fling you” heard as an echo of being thrown off a bus by a school bully. The immediacy of dialogue (delivered within a safe, controllable context) often cued high affect, accessing the heart of meaning-making (“hotspots”). Self-compassion could be particularly challenging for survivors of sexual abuse.^18^ However, powerful statements did emerge spontaneously in dialogue (reinforced by the avatar response “I never thought I’d hear you say that.”)

### Future Focus

The final session involved future planning guided by the personal meaning of recovery.^19^ Some voice-hearers expressed sadness about saying goodbye to the avatar; the person was supported to acknowledge what the avatar/voice had represented while committing to reclaiming power and control in their life.

## Acceptability of AVATAR Therapy

Of the 75 people randomized to receive therapy in the recent trial,^4^ 29% (*n* = 22) withdrew at some stage from therapy (Table 3). Of the 17 who withdrew having created their avatar, only eight gave reasons explicitly related to AVATAR therapy. All withdrawals occurred within phase 1, suggesting exposure work needs to be approached sensitively. One withdrawal related to an early change in the avatar from hostile to conciliatory, that the person experienced as out of keeping with how their voices would respond, prompting us to consider the risk of invalidation during the transition. Once people had overcome the initial exposure to anxiety, no withdrawals occurred within phase 2, indicating that engagement in elaborated dialogue was acceptable. There were no adverse events attributable to AVATAR therapy.^4^ Voice-activity presented an issue for a small minority, however, most reported voices as less active during active dialogue. One person reported seeing his avatar reflected in a shop window between sessions, but described this as a benign, comforting experience. Some reported voices commenting on therapy or discouraging attendance. They were supported to address this directly with the avatar.

**Table 3.** Withdrawals Including Timing and Relatedness to AVATAR Therapy (from Overall *n* = 75^4^)

Timing	*N*	AT-related	Reasons
Pretherapy	5	0	—
Assessment/ creation	7	4	Found creating the avatar distressing Not keen on discussing the past Found avatar anxiety provoking “Too much for me at this point”
After session 1	5	1	“Did not think it would be helpful”
After session 2	2	1	“Approach is not for me”
After session 3	3	2	“Didn’t like it when the avatar turned nice/ said sorry” (avatar transition) Felt approach was not helpful for him
Sessions 4–6	0		—
Total	22	8	

## Discussion

We have presented, for the first time, a comprehensive account of AVATAR therapy, illustrating the full range of therapeutic targets across two phases of therapy. In this study, standardized booklets provided direct therapist self-report on session-by-session therapy delivery. The systematic review conducted by the two people responsible for close supervision of all therapy on the trial (delivering the majority of therapy personally) was considered the most clear and valid way of answering the crucial question: What are therapists targeting in the delivery of AVATAR therapy? However, it should be acknowledged that alternative methods of analyzing complex psychological interventions would be required to address other important questions, eg, qualitative analysis of voice-hearers experience of therapy and linguistic analysis of specific dialogic exchanges.

### Potential Challenges Relating to AVATAR Therapy

AVATAR therapy is multifaceted and requires experienced therapists. It involves exposure to raw and painful experiences that the voice-hearer may have never shared. Phase 1 can be particularly challenging for the person and therapist. However, with high levels of self-reported verisimilitude voice-hearers frequently reported a sense of confidence, achievement, and liberation through dialogue, demonstrating courage and resilience in ways that clinicians can, at times, overlook.

AVATAR therapy presents delivery challenges (eg, switching between speaking as therapist and avatar in real-time) and ethical considerations for therapists who voice abuse (through the voice-transformed avatar) and reenact critical and abusive relationships. Clinicians should be mindful of the power of this approach and sensitive to emotionally loaded content (especially involving abuse and discrimination). Direct work with verbatim content provides an important opportunity for the voice-hearer to reclaim power over words previously used to silence and disempower. Therapy delivery requires skill, sensitivity, and effective training and supervision. The voice-hearer’s understanding, existing support and readiness for this approach should be assessed and regularly reviewed. The therapeutic relationship is to the fore, with an emphasis on respect and collaboration. The potential for past experiences to “bleed into” the relationship must be considered in supervision with close attention to the operation of power.

Some might question whether AVATAR therapy unhelpfully positions the voice as an “adversary to be banished” in contrast to other approaches.^3^ However, the way in which the avatar is enacted is grounded in how the voice is understood and experienced by the person. Some came to AVATAR therapy with an expressed hope to “get rid” of their voices. For others, the voice represented their main social connection; voice-relationships can offer meaning and purpose and an escape from tedium and loneliness. The focus is always on what is distressing and interfering with life.^20^ Some voices (including hostile and abusive ones) were ascribed a positive communicative intention (eg, “toughening up” or “protection”). Developing compassion toward the voice represents a positive endpoint for many voice-hearers and featured explicitly in around a quarter of the cases. Likewise, acceptance of the voice as “part of me” ^19^ was transformative for some. However, what voice-hearers identify as positive change can vary. Differential pathways connecting early trauma and later phenomenology,^17^ as well as possible voice subtypes^21^ suggest that a focus on positive function may carry more therapeutic value for some voices than others. Our approach is for the dialogue to evolve with the person in control of the changing relationship, and to enable increased power to generalize to the everyday voice experience. An analogy can be made to relationships of domestic violence characterized by coercion and control. Abusers often silence the person in ways that are mirrored in voice-hearing (“if you tell anyone I’ll kill you”). Some voice-hearers found power in calling the abuser to account. Compassion and acceptance are always on the table. However, the opportunity to express “righteous anger” and to dismiss the abuser can be liberating. Indeed, it can be the start of relinquishing shame and self-blame, sowing the seeds of burgeoning self-compassion.

### Future Directions

AVATAR therapy is at a relatively early stage of development and dissemination. Substantial early improvements were reported for some, posing the question of whether phase 1 could be sufficient in instances where distress is closely linked to fear, anxiety, and relational submissiveness. Furthermore, certain forms of voice-hearing may be particularly amenable to AVATAR therapy (and relational work more generally) with voice “characterization” particularly prominent during phase 2. Future work will focus on further personalizing and optimizing therapy delivery and evaluating effectiveness with a view to dissemination. AVATAR dialogue can be considered a unique therapeutic crucible allowing dynamic self-self and self-other representation to be enacted in vivo alongside core emotional processes, suggesting transdiagnostic potential; work is underway in the application of AVATAR therapy to the “anorexic voice.”

## Conclusion

For many who hear distressing voices, the experience is fundamentally social, involving communication with a characterized “other.” Abuse and intimidation are drip-fed into relationships of dominance, coercion, and control; crystallizing into fear, shame, and powerlessness. AVATAR therapy offers a powerful therapeutic context, involving exposure to an embodied representation of the disembodied voice and direct real-time work on “hot” cognitive, emotional, and relational processes. We have presented a comprehensive account of AVATAR therapy, identifying for the first time the full range of therapeutic targets. The aim of AVATAR therapy is to enable the person to (re)build a sense of power, control, and self-respect; enacting new modes of social relating that they carry into their life. This article represents a touchstone for development in this important approach.

## Funding

The research was funded by the Wellcome Trust (FWBC-AVATAR 098272/z/12/z). P.G. and T.K.J.C. were part-funded by the National Institute for Health Research (NIHR) Biomedical Research Centre at South London and Maudsley NHS Foundation Trust and King’s College London. The views expressed are those of the author(s) and not necessarily those of the NHS, the NIHR or the Department of Health.
